# Immunomodulatory Effects of Dietary Polyphenols

**DOI:** 10.3390/nu13030728

**Published:** 2021-02-25

**Authors:** Hira Shakoor, Jack Feehan, Vasso Apostolopoulos, Carine Platat, Ayesha Salem Al Dhaheri, Habiba I. Ali, Leila Cheikh Ismail, Marijan Bosevski, Lily Stojanovska

**Affiliations:** 1 Department of Nutrition and Health, College of Medicine and Health Sciences, United Arab Emirates University, Al Ain 15551, United Arab Emirates; 201890012@uaeu.ac.ae (H.S.); PlatatCarine@uaeu.ac.ae (C.P.); ayesha_aldhaheri@uaeu.ac.ae (A.S.A.D.); habali@uaeu.ac.ae (H.I.A.); 2 Institute for Health and Sport, Victoria University, Melbourne 3011, Australia; jfeehan@student.unimelb.edu.au (J.F.); vasso.apostolopoulos@vu.edu.au (V.A.); 3 Department of Medicine-Western Health, The University of Melbourne, Melbourne 3000, Australia; 4 Clinical Nutrition and Dietetics Department, College of Health Sciences, Research Institute of Medical and Health Sciences (RIMHS), University of Sharjah, Sharjah 27272, United Arab Emirates; lcheikhismail@sharjah.ac.ae; 5 Nuffield Department of Women’s & Reproductive Health, University of Oxford, Oxford OX1 2JD, UK; 6 St. Cyril and Methodius Faculty of Medicine, University Cardiology Clinic, 1000 Skopje, North Macedonia; marijanbosevski@yahoo.com

**Keywords:** polyphenols, immunomodulation, pro-inflammatory cytokines, anti-inflammatory cytokines

## Abstract

Functional and nutraceutical foods provide an alternative way to improve immune function to aid in the management of various diseases. Traditionally, many medicinal products have been derived from natural compounds with healing properties. With the development of research into nutraceuticals, it is becoming apparent that many of the beneficial properties of these compounds are at least partly due to the presence of polyphenols. There is evidence that dietary polyphenols can influence dendritic cells, have an immunomodulatory effect on macrophages, increase proliferation of B cells, T cells and suppress Type 1 T helper (Th1), Th2, Th17 and Th9 cells. Polyphenols reduce inflammation by suppressing the pro-inflammatory cytokines in inflammatory bowel disease by inducing Treg cells in the intestine, inhibition of tumor necrosis factor-alpha (TNF-α) and induction of apoptosis, decreasing DNA damage. Polyphenols have a potential role in prevention/treatment of auto-immune diseases like type 1 diabetes, rheumatoid arthritis and multiple sclerosis by regulating signaling pathways, suppressing inflammation and limiting demyelination. In addition, polyphenols cause immunomodulatory effects against allergic reaction and autoimmune disease by inhibition of autoimmune T cell proliferation and downregulation of pro-inflammatory cytokines (interleukin-6 (IL-6), IL-1, interferon-γ (IFN-γ)). Herein, we summarize the immunomodulatory effects of polyphenols and the underlying mechanisms involved in the stimulation of immune responses.

## 1. Introduction

**Figure 1:**
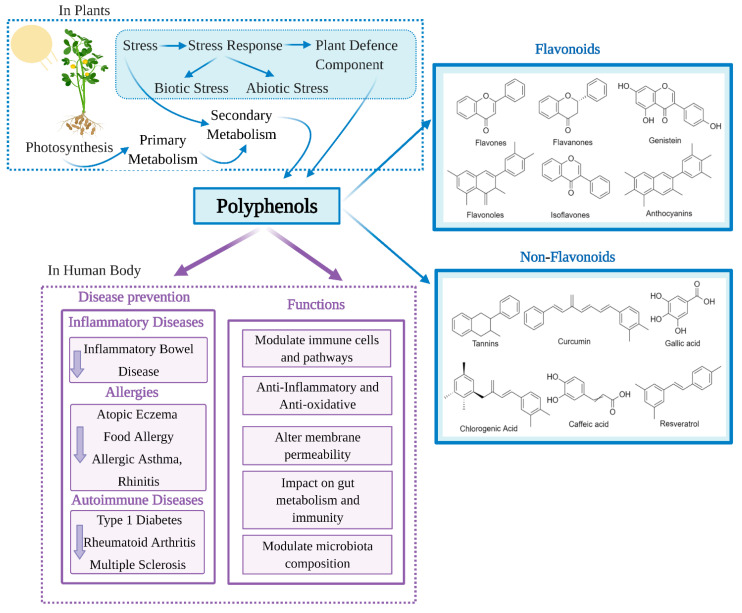
Classification and health benefits of polyphenols.

With advancing knowledge of the importance of adequate nutrition, and increased public health awareness about diet, there is growing attention on the health benefits of natural products including those that are rich in polyphenols. Polyphenols are the most extensive group of non-energetic secondary metabolites and are produced by plants in response to stress [1] (Figure 1). Polyphenols have been called ‘lifespan essentials’ due to their significant impact on health [2]. There are as many as 8000 different polyphenols which are divided into different classes based on their chemical structure. Despite the different classifications, all polyphenols have the key structural features of an aromatic ring and at least one hydroxyl group [3,4]. Dietary polyphenols are abundant in plant-based foods such as fruits, vegetables, dry legumes, cereals, olives, cocoa, tea, coffee and wine [5]. Some common dietary polyphenols include the lignins present in nuts and whole-grain cereals; pro-anthocyanidins in grapes, pine bark and cocoa; anthocyanins/anthocyanidins in brightly colored fruits and vegetables like berries; isoflavones in soybeans; catechins in green tea, grapes and wine; tannins in tea and nuts; quercetin in grapes and onion; resveratrol in wines and naringenin/hesperidin in citrus fruits [6].

Research into the beneficial health effects of polyphenols has increased considerably over the last two decades [7]. Polyphenols have shown anti-inflammatory, antimicrobial, antioxidant, anticarcinogenic, antiadipogenic, antidiabetic and neuroprotective effects [8,9,10,11,12]. Polyphenols may also counteract cytotoxicity and apoptosis due to their immunomodulatory properties [13] and regulate innate and adaptive immunity. Polyphenols have also been shown to reduce oxidative stress and inflammation [14], modulate immune cells, regulate gut microbiota composition and immunity (Figure 1). Through this regulation of the immune system, polyphenols could beneficially impact a number of chronic diseases [15]. Herein, we discuss the immunomodulatory effect of polyphenols and the resulting effects on different chronic diseases, including inflammatory bowel disease, atopic eczema or dermatitis, allergic asthma, rhinitis, type 1 diabetes, multiple sclerosis and rheumatoid arthritis.

## 2. Methods

Extraction of current and relevant data was performed using the electronic databases, Science Direct, PubMed, Springer and Google Scholar. Searches were conducted in two sections. The first part aimed to identify evidence on the effect of polyphenols on the immune system and immune cells. Search terms used were ‘Polyphenols’ OR ‘Phytochemicals’ OR ‘Phenolic’ AND ‘Immunity’ OR ‘Immune system’ OR ‘Immune function’ AND ‘Immune cells’ OR ‘Dendritic cells’ OR ‘Macrophages’ OR ‘Monocytes’ OR ‘Neutrophils’ OR ‘Natural Killer cells’ OR ‘B cells’ OR ‘T cells’ OR ‘T helper cells. The second part aimed to identify evidence on the impact of polyphenols in chronic inflammatory and auto-immune diseases. Additional search terms included ‘Inflammatory diseases’ OR ‘Inflammatory Bowel Disease’ AND ‘Allergy’ OR ‘Atopic Eczema’ OR ‘Dermatitis’ OR ‘Food Allergy’ OR ‘Rhinitis’ OR ‘Asthma’ AND ‘Autoimmune Disease’ OR ‘Type-1 diabetes’ OR ‘Rheumatoid arthritis’ OR ‘Multiple sclerosis’. Articles published in English were included. The titles and abstracts were scanned to exclude any irrelevant studies. A total of 167 papers that focused only on the immunomodulatory effect of polyphenols on health were screened and articles containing relevant data were reviewed.

## 3. Immune Modulation of Polyphenols to Immune Cells

**Figure 2:**
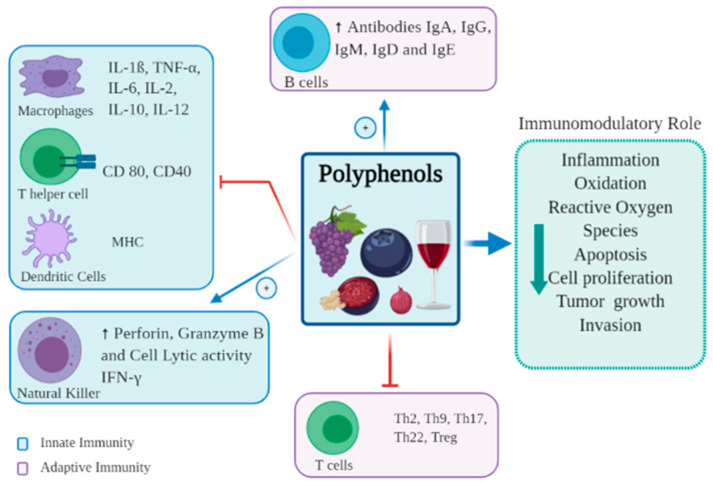
Immunomodulatory effects of polyphenols on immune cells.

The immune system as a whole consists of innate and adaptive immunity, each with different roles and functions [16]. The innate immune system is the first line of defense, and protects against foreign antigens through the skin, pulmonary system, and gut epithelial cells, forming a barrier between the organism and its environment [17]. The innate system is broadly divided into cellular and non-cellular systems. The cellular system consists of several cell subsets, including dendritic cells (DCs), monocytes, macrophages, granulocytes and natural killer (NK) cells. The non-cellular system is very diverse, ranging from simple mucus barriers to complex protein pathways, such as the complement cascade, however all function to prevent pathogen entry, and facilitate pathogen destruction by phagocytosis [18]. The adaptive immune system comprises T and B cells. B cells secrete antibodies, whilst T cells are involved in the production of cytokines, direct cytotoxic destruction of infected or malignant tissue, and activation of other immune cells [16]. Polyphenols modulate immune responses in both the innate and adaptive systems, having both stimulatory and inhibitory effects in different areas [19] (Figure 2).

### 3.1. Effects of Polyphenols on Dendritic Cells

DCs are the most potent antigen-presenting cells which act to prime the adaptive immune system to recognize foreign antigens, and so are vital in the initiation and regulation of the adaptive immune response [20]. It has been shown that polyphenols can influence the differentiation of DCs [21]. In fact, resveratrol has been identified as affecting human DC differentiation from monocytes, with a strong potential for regulatory action [22]. Likewise, epigallocatechin gallate (EGCG) induces apoptosis and affects the phenotype of developing DCs. Molecules that are essential for antigen presentation by DCs such as CD83, CD80, CD11c, and major histocompatibility complex (MHC) class II, are downregulated by EGCG, suggesting an immunosuppressive action [23]. Other polyphenols, including EGCG, curcumin, quercetin, apigenin, silibinin, and blackberry polyphenols cause inhibition of murine bone marrow-derived DC maturation and expression of MHC molecules, reducing antigen uptake and decreasing secretion of the pro-inflammatory cytokines interleukin-1 (IL-1), IL-2, IL-6, IL-12 [24,25,26,27]. A study in an animal model showed that administration of fisetin (50 mg/kg) decreased DC migration and DC allo-stimulatory capacity [28]. Similarly, in vitro resveratrol has an inhibitory effect on DC maturation [29].

### 3.2. Effects of Polyphenols on Monocytes and Macrophages

Macrophages are phagocytes that ingest pathogens and dead cells, which differentiate from the transitory monocyte. Like DCs, macrophages can also function as antigen-presenting cells (albeit with less potent activity) being able to activate naïve T cells into effector T cells in the presence of an antigen [19]. Macrophages play an important role in inflammation, host defense, and tissue repair [30,31]. Importantly, macrophages also play a pathogenic role in various chronic diseases including asthma, inflammatory bowel disease, atherosclerosis and rheumatoid arthritis [31,32,33]. Macrophages are classically considered in two categories, known as polarization: the classical inflammatory M1 and immunosuppressive/anabolic M2 phenotypes. Initiation of M1 differentiation is by interferon-γ (IFN-γ) stimulation and the activation of toll-like receptors (TLRs) by bacterial lipopolysaccharides (LPS); while M2 polarization is triggered by IL-4 [34]. It has been shown that polyphenolic cocoa extract suppressed M1 mediated inflammation and drove M2 polarization of activated macrophages [35]. Polyphenol-rich green tea has anti-tumor effects secondary to the activation of macrophages and NK cells [36]. Inonotus sanghuang, a plant known for its medicinal value, rich in rutin, quercetin, quercitrin, isorhamnetin and chlorogenic acid, has been shown to reduce inflammation by modulating the interaction between macrophages and adipocytes. It was suggested that in this way it may improve insulin resistance and metabolic syndrome [37]. Moreover, Overman et al. reported that grape powder extract decreased LPS-stimulated inflammation in macrophages and reduce insulin resistance [38].

Monocytes and macrophages play a fundamental role in the progression of atherosclerosis [35]. Increased oxidative stress causes low-density lipoprotein oxidation (oxLDL), with the resulting lipoproteins engulfed by macrophages resulting in the formation of foam cells. This process triggers an inflammatory response in the neighboring endothelial cells which secrete pro-inflammatory cytokines and chemokines [39,40,41]. When monocytes migrate towards the intima, they transform into macrophages on stimulation by macrophage colony-stimulating factor, increasing the expression of scavenger receptors outside the cell [39,40]. Polyphenols are known to regulate this interplay between immune and vascular endothelial function. Evidence has shown that polyphenols reduce atherosclerotic progression by increasing high-density lipoprotein (HDL) levels and decrease LDL accumulation in macrophages, reducing foam cell formation [3,42].

### 3.3. Effects of Polyphenols on Natural Killer Cells

NK cells are a subset of lymphocytes, but are part of the innate immune response, with the function of eliminating infected or malignant cells [19]. NK cells have a strong cytolytic function and a considerable role in immune regulation [43]. NK cells are activated by CD4+ T cell secretion of IL-2 and IFN-γ [44]. Once activated NK cells secrete perforin and granzyme B, which induce apoptosis and necrosis in target cells. Polyphenols have immunomodulatory effects on NK cells, increasing their number and activity. Green tea catechin metabolites increase NK cell cytotoxicity [45] and quercetin enhances NK cell lytic activity [46] in animal models. In a clinical trial, healthy participant prescribed a diet low in polyphenols and supplemented with juices rich in polyphenols increased lymphocyte proliferative responsiveness, IL-2 secretion and lytic activity by NK cells [47]. Berries rich in flavonoids and pro-anthocyanidins have a cancer-preventive effect but are also involved in the modulation of NK cells [48]. A study in marathon runners noted that daily consumption of 250 g of blueberries for six weeks resulted in doubled NK cell counts [49]. Evidence showed that purple sweet potato leaves that are rich in flavonoids enhanced the lytic activity of NK cells in 16 healthy participants [50].

### 3.4. Effects of Polyphenols on T and B Cells

T and B cells are the principal components of the adaptive immune system. B cells secrete antibodies known as immunoglobulins (Igs), which bind to antigens and underpin hypersensitivity reactions and antimicrobial immune responses. When B cells are activated to a specific antigen, they differentiate into plasma cells and produce Immunoglobulin (Ig)A, IgG, IgM, IgD and IgE [19]. Polyphenols have been suggested to modulate the function of B cells; however, this has been poorly described, and further research is required. In an in vitro study it was noted that green tea polyphenols and EGCG decreased the production of IgE in a dose and time-dependent manner [51], and another study showed that polyphenols inhibit the proliferation of CD19+ cells and reduce IgG production [52]. It was noted that administration of green tea extract to mice for 6 weeks reduced the production antigen-specific IgE by enhancing CD4+ CD25+ regulatory T cells (Treg) in the spleen, resulting in reduction of allergic response [53].

T cells are divided into three major types: cytotoxic T cells, T helper (Th) cells and the Treg cells depending upon expression of the CD4 or CD8 molecules. CD4+ T cells are helper T cells that assist and control immune cell activity and activation. CD8+ cytotoxic T cells act to directly lyse and destroy malignant, senescent or infected cells [19]. Polyphenols have been associated with the modulation of enzymatic signaling, via the inhibition of the serine-threonine and tyrosine-protein kinase pathways. These enzymes are primarily linked with B cell activation and T cell proliferation as well as the production of cytokines by activated monocytes [3]. Treg play a crucial role in immunity tolerance and control of auto-immunity [54]. A study on mice showed that regular treatment of EGCG for a week increased the frequency of Treg cells in the spleen, pancreatic lymph nodes and mesenteric lymph nodes. The Treg cells obtained from the treated group could suppress cytotoxic T cell function, reducing proliferation and IFN-γ production [54]. In addition, EGC-M5 (a major metabolite of EGCG) at a dosage of 10 mg/kg of body weight were provided to rats for 14 days and caused upregulation of CD4+ T cell activity and cytotoxic activity of NK cells [45].

### 3.5. Effects of Polyphenols on T Cell Differentiation

CD4+ T helper cells differentiate into T helper (Th)1, Th2, Th9, Th17, Th22, depending upon the cytokine environment [55]. Th1 cells are involved in cell-mediated immunity and are produced in the presence of IL-12, whilst Th2 cells are critical for humoral immunity and differentiate in the presence of IL-4 and IL-13 [56]. Th17 cells secrete IL-17, IL-22, and chemokine ligands 20 (CCL20) [57] and have been shown to have a role in the progression and pathogenesis of chronic inflammatory diseases like rheumatoid arthritis, multiple sclerosis, psoriasis, atopic dermatitis, and asthma [56,58]. Th22 cells produce IL-22, a cytokine responsible for maintaining the epithelial barrier and skin integrity [57]. In mice, polyphenols like apigenin and chrysin suppresses serum IgE induced by ovalbumin immunization by downregulating Th2 responses [59]. Similarly, tea polyphenols, such as EGCG, reduce Th1 differentiation and numbers of Th17 and Th9 cells [60], as well as resveratrol by decreasing Th17 cell numbers in an inflammatory arthritis model in rodents [61]. Grape seed pro-anthocyanidin extract also showed anti-arthritic properties and upregulated the number of Tregs and maintained the balance between Th17/Treg, attenuating inflammation [62].

### 3.6. Effects of Polyphenols in Inflammation

The inflammatory response of the innate immune system is a vital part of the defense against microbial infection. However despite its vital role in promoting the immune response, its timely resolution is equally important [63]. Chronic inflammation is a key cause of a number of life-threatening diseases [64]. A study on pomegranate peel polyphenols (PPPs) and its specific components such as punicalagin (PC) and ellagic acid (EA) showed a reduction in pro-inflammatory cytokines TNF-α, IL-1β and IL-6 and downregulation other inflammatory mediators including nitric oxide (NO) and prostaglandin E2 (PGE_2_) by decreasing inducible nitric oxide synthase (iNOS) and cyclooxygenase-2 (COX-2) expression [65]. Similarly, PPPs, PC, and EA have shown inhibitory effects on LPS-induced production of intracellular reactive oxygen species (ROS) and suppression of TLR4 at both the mRNA and protein levels, all of which have major mechanistic roles in inflammation [66]. In addition, grape seed extract, has been shown to decrease pro-inflammatory cytokine, ROS and superoxide production whilst elevating antioxidant enzyme gene expression and secretion of anti-inflammatory mediators [67,68]. Green tea polyphenols also reduce the production of inflammatory cytokines (TNF-α, IL-6, IL-1β), and inhibit the TLR4 signaling pathway [69]. The immunomodulatory effects of polyphenols are summarized in Table 1.

## 4. Immune Modulation of Polyphenols to Prevent and Control Chronic Diseases

Dietary polyphenols have preventive and therapeutic potential for a number of chronic diseases whose development involves dysregulation of the immune function.

### 4.1. Polyphenols and Inflammatory Bowel Disease

The intestinal epithelium is generally in a state of low-grade inflammation, due to microbial, chemical and mechanical stimuli that maintain a moderate inflammatory response [80]. Generally, it is controllable, but if inflammation exceeds the normal limit due to disease, it can disrupt epithelial tissues and impede intestinal dysfunction. These uncontrollable inflammatory conditions are known collectively as inflammatory bowel disease (IBD), comprised of two specific pathologies; Crohn’s disease and ulcerative colitis [81]. Globally, the annual incidence of IBD is approximately 396 cases per 100,000 persons per year [82]. There is evidence to suggest polyphenol supplementation could play a role in managing IBD. The proposed mechanism by which this occurs is through polyphenol modulation of pattern recognition receptors (PRRs), such as the TLRs and nucleotide-binding oligomerization domain proteins, which are highly expressed in intestinal epithelial and immune cells. PRRs activate immune responses against pathogens through recognition of related molecular structures [83], and polyphenols are known to be able to modulate the expression of PRRs and their associated inflammatory response in the intestine [80]. Activation of PRRs induces inflammation by increasing cytokine secretion and cyclooxygenase-2 expression. Polyphenols like curcumin and isothiocyanate inhibit TLR4 dimerization, an essential step for TLR4 activation [84,85]. Resveratrol also interferes with TLR4 signal transduction by inhibiting TANK binding kinase 1 which regulates the downstream pathways that result in cytokine production [86]. In addition, resveratrol acts as anti-inflammatory agent in intestinal mucosa [87]. Polyphenols are also known to modulate key inflammatory genes, such as cyclooxygenase-2 and the inflammatory cytokines, further implying potential for an anti-inflammatory effect in IBD [88,89]. It has also been shown that flavonoids are able to regulate the activity of Treg cells in the intestine, downregulating the expression of inflammatory cytokines, and consequently suppressing inflammation [90,91]. Polyphenols are also able to influence the gut microbiota as a probiotic. Green tea polyphenols promote the growth of beneficial microbiota like *Bifidobacterium* and *Lactobacillus* and suppress pathogenic bacteria, such as, *E Coli* and *Salmonella* [12,92]. This supports the maintenance of intestinal homeostasis and reduces inflammation [93]. Grape seed and green tea polyphenols also have potential to prevent or delay the progression of IBD [68,94,95]. Pomegranate polyphenols also provide protective effects against IBD by modulating the intestinal inflammatory response reducing expression of various pro-inflammatory cytokines, such as iNOS, COX-2, PGE2, as well as regulating the composition of the luminal microbiota [96]. A recent study reported that dietary polyphenols from mango (gallotannins and gallic acid) improved the symptoms of IBD. This study included 10 subjects who received 200–400 g/day of mango pulp for 8 weeks. A significant reduction was observed in a factors related to neutrophil-induced inflammation like IL-8, growth-regulated oncogene and granulocyte macrophage colony-stimulating factor by 16.2%, 25.0% and 28.6%, respectively [97]. Another study showed that Bronze tomatoes, which are rich in anthocyanins, flavonols, and stilbenoids, had a significant impact in alleviating the symptoms of IBD in mice [98]. Taken together, this suggests that polyphenols can help in the prevention and treatment of IBD by reducing pro-inflammatory cytokines, regulating the activity of Treg cells and promoting the growth of beneficial microbiota in the intestine.

### 4.2. Polyphenols and Allergies

The prevalence of allergic disorders has been increasing dramatically with competing environmental, genetic, diet and hygiene factors likely to underlie their advance [99,100]. Allergic reactions result from a hyper-reaction of the immune response against allergens such as those in the environment (dust, grass pollen) or food (milk, fish, eggs, nuts and wheat) [101]. Due to their growing incidence, there is significant attention on interventions to assist in their management, and polyphenols have been proposed as viable solutions [101] (Table 2). Certain polyphenols influence allergic responses at two critical stages: (1) allergic sensitization and (2) re-exposure to the allergen. During the sensitization phase, polyphenols such as caffeic and ferulic acid bind with allergenic proteins, forming insoluble complexes and rendering them non-reactive [101]. Additionally, flavonoids directly affect antigen presentation by DCs by either inhibiting cell surface expression of MHC-II and co-stimulatory molecules (CD80, CD86), leading to ineffective antigen presentation, or by inhibiting cytokine production [102,103]. Polyphenols like catechins and their derivatives inhibit Th2 cytokine production [104,105] as well as T cell activation and proliferation [106,107]. Recruitment of B cells to sites of allergic inflammation and their production of IgE have also been shown to be inhibited by polyphenols [59,108,109]. Of interest, the interaction between polyphenols and proteins results in the modulation of allergic sensitization and their direct effect on mast cells hence inhibiting the release of allergic mediators and eventually decreasing the symptoms of allergy [101]. In addition, polyphenols such as caffeic, ferulic, and chlorogenic acids can bind irreversibly with the peanut allergens, Ara h1 and Ara h2, reducing their allergenicity [110]. In mice, administration of polyphenol-enriched areca nut extracts suppressed the level of ovalbumin (OVA)-specific IgE, the expression of IL-4, downregulating Th2 driven immunity and enhancing the activity of myeloid-derived suppressor cells, attenuating allergic responses [111]. In another study, 30 female mice treated with cranberry and blueberry polyphenol complexed peanut protein for 6 weeks, had reduced expression of CD63 and decreased plasma IgE levels [112]. Evidence has also shown that polyphenol-rich cranberry extracts interact with wheat gliadins forming insoluble complexes in a mouse model, which decreased wheat gliadin immunogenicity and allergenicity [113]. Furthermore, polyphenolic ellagic acid effectively binds with allergenic proteins within the food matrix [114,115]. Punicalagin (a polyphenol derived from pomegranate), rutin and phloridzin increased the growth of beneficial bacteria species such as *Bifidobacterium* and *Lactobacillus* which are known to have beneficial impacts in food allergies [116,117]. Oral administration of a polyphenol-rich grape skin extract that had been fermented with *Lactobacillus Plantarum* had an inhibitory effect on allergic responses when compared to a non-fermented extract [118].

### 4.3. Polyphenols in Atopic Eczema or Dermatitis

Atopic eczema and dermatitis are allergic skin disorders that primarily occur during early infanthood (3–4 months of age) and continue to develop until 2 years of age [128]. They cause dryness of skin, itching, inflammation and erythema (redness). Polyphenols have anti-inflammatory properties that can alleviate this allergic inflammation. Green tea extracts (catechins, epicatechin, epigallocatechin gallate and their derivatives) protect against cutaneous inflammation [129]. Similarly, EGCG suppresses the secretion of the pro-inflammatory cytokine IL-2 in vitro, an important mediator in allergic dermatological conditions [130]. Polyphenols have also been shown to improve the characteristic itching and pruritis associated with these conditions. Oat-derived polyphenol avenanthramide was shown to reduce the characteristics of pruritis and itching associated with dermatological conditions [131]. The effect of polyphenols on keratinocytes and immune cells was also analyzed in vitro and shown to reduce nuclear factor κB (NF-κB) activation, TNF-α and IL-8 [132]. Likewise, quercetin and luteolin also inhibit skin itching and flush reaction [133]. By suppressing pro-inflammatory cytokines, polyphenols can reduce the symptoms and occurrence of allergic skin disorders.

### 4.4. Polyphenols in Allergic Asthma and Rhinitis

Asthma, an allergic inflammatory lung disease characterized by increased leukocyte infiltration into the airways, most notably granulocytes, resulting in decreased respiratory function. Often the inflammation can cause bronchoconstriction, airway hyper-responsiveness (AHR) and increased mucus production [134]. In the airways, exposure to an allergen such as pollen produces a Th2-dominated response by recruiting and activating inflammatory cells and upregulating IL-4, IL-13, and IL-5 [135]. In an animal model of asthma, it was shown that resveratrol had a suppressive effect on asthmatic parameters as it inhibited the production of Th-2 cytokines like IL-4 and IL-5 in the plasma and bronchoalveolar lavage fluid, and caused suppression of airway hyperresponsiveness, eosinophilia, and hypersecretion of mucus [136]. Moreover, quercetin is known to ameliorate the pathogenic process of asthma by decreasing IL-4 and IFN-γ synthesis and by regulating Th1/Th2 balance [137]. Recent evidence showed that the administration of polyphenol-rich ethanolic extract of *Boehmeria nivea* (caffeic acid, catechin, epicatechin, β-sissterol, rutin, luteolin-7-glucoside, naringin, hesperidin, chlorogenic acid, and tangeretin) reduces allergic response in mice by suppressing mast cell mediated inflammation, decreasing TNF-α, IL-1β, IL-6, Th2, extracellular-signal-regulated kinase (ERK) and Mitogen-Activated Protein Kinase (MAPK) expression [138]. In murine asthma models, it was found that sesamin (rich in flavonoids) reduced allergic inflammation induce by asthma and airway hyperresponsiveness (AHR), making them an effective adjunct for the treatment of asthma [139].

## 5. Immune Modulation of Polyphenols in Autoimmunity

The immune system protects against foreign substances, but it is also responsible for self-tolerance, by which host tissues are protected from immunological action. Dysfunction can lead to loss of this immune tolerance and disturbed homeostasis, resulting in autoimmune disease [140]. The prevalence of autoimmune diseases is about 5%, and approximately 80 types of autoimmune diseases have been described [141]. Several factors lead to the development of autoimmune diseases, including genetic, epigenetic, environmental, nutritional and microbiotic diseases. Polyphenols have been shown to have a beneficial role in some common autoimmune diseases.

### 5.1. Polyphenols and Type-1 Diabetes

Type-1 diabetes is a multifactorial disease linked to a combination of genetic and environmental factors. It is characterized by the autoimmune destruction of pancreatic β cells, resulting in severe insulin deficiency and resultant hyperglycemia [142]. Polyphenols help in the regulation of pancreatic β-cells, type-1 diabetes and complications associated with type-1 diabetes [143]. Polyphenols involved in activation of the phosphatidylinositol 3-kinase/protein kinase B (PI3K/Akt) signaling pathway, thus helping to reduce the progression of type-1 diabetes [140]. It was shown that pomegranate peel extract inhibited immune cell infiltration into pancreatic islets and interferes with IL-17 and IFN-γ synthesis in gut-associated lymphoid tissue in type-1 diabetes [144]. A study in mice with type-1 diabetes quercetin treatment modulated Th1/Th2 balance and had glucose-lowering potential [145]. In addition, butein (a plant polyphenol) was able to prevent cytokine-induced β-cell damage by inhibiting NO production, iNOS expression, NF-κB translocation and glucose-stimulated insulin secretion which, prevented the progression of type-1 diabetes in rats [146]. Similarly, *Broussonetia kazinoki* polyphenols have been shown to have therapeutic potential in the prevention of cytokine-induced β-cell damage and reduce/delay the extent of pancreatic β-cell damage in type-1 diabetes [147]. Consequently, polyphenols may play a role in modulating key signaling pathways, T helper cell response and reducing cytokine induced β-cell damage, which may aid in the management of type-1 diabetes.

### 5.2. Polyphenols and Rheumatoid Arthritis

Rheumatoid arthritis, is a systemic autoimmune disease, characterized by erosive and symmetric synovitis, particularly in the peripheral joints. Degradation of cartilage and bone erosion leads to the eventual destruction of a joint [148]. In developed countries, rheumatoid arthritis affects about 0.5–1% of the population and women are at three times greater risk [149]. While the reason for the development of this disease is still unknown, genetics is thought to play an important role. Polyphenols may also have an impact in the management of rheumatoid arthritis. For instance, curcumin, a potent anti-inflammatory agent, decreases IL-1β, induces IL-6 and vascular endothelial growth factor by rheumatoid arthritis-fibroblast-like synoviocytes [150]. In addition, curcumin also induces apoptosis of rheumatoid arthritis-fibroblast-like synoviocytes, which are typically resistant to apoptotic signaling, by upregulating pro-apoptotic proteins, such as Bax, and downregulating the anti-apoptotic protein Bcl-2 [151]. In addition, resveratrol has both protective and therapeutic effects in inflammatory arthritis, by inhibiting the function of Th-17, B-cells and MAPK signaling pathways [61,152]. A clinical trial reported that a 1 g capsule of resveratrol for 3 months decreased the swelling and tenderness of joints by regulating pro-inflammatory cytokines [153]. Moreover, EGCG suppresses osteoclast differentiation and decreases clinical symptoms in an animal model of rheumatoid arthritis [154]. Grape polyphenols have shown immuno-regulatory effects, establishing a balance between Th17 and Treg cells and inhibiting TNFα in rheumatoid arthritis, hence mitigating inflammation, oxidative stress and rheumatoid arthritis associated symptoms [155,156,157]. Administration of extra virgin olive oil polyphenol extract (100 and 200 mg/kg body weight) to arthritic mice decreased the pro-inflammatory cytokines, PGE2, COX-2, and microsomal prostaglandin E synthase-1 as well as NF-κB translocation, resulting in decreased progression of the joint disease [158]. Evidence showed that quercetin supplements (500 mg/day) for 8 weeks resulted in a significant reduction of morning stiffness, morning pain, and after-activity pain [159]. Therefore, polyphenols may improve the quality of life of patients with rheumatoid arthritis.

### 5.3. Polyphenols and Multiple Sclerosis

Multiple sclerosis, is a chronic neurological autoimmune disorder characterized by the breakdown of the myelin sheath, alongside dysfunction of the blood–brain barrier, perivascular inflammation, as well as damaged axons and oligodendrocytes-all of which lead to progressive nervous system damage and clinical disability [140]. Polyphenols may play a role in the prevention and treatment of multiple sclerosis [160]. Quercetin exerts an immunomodulatory effect aiding in the treatment of multiple sclerosis by inhibiting proliferation of autoimmune T-cells, and the expression of TNF-α by mononuclear cells in vitro [161,162]. It has also been shown to reduce peripheral blood mononuclear cell proliferation [163]. The polyphenolic flavones apigenin and luteolin have a robust inhibitory potential on T-cell proliferation while also reducing IFN-γ production [162]. Strikingly, flavonoids have been shown to limit demyelination in multiple sclerosis and so may confer neuroprotective benefits [164]. In a mouse model, resveratrol prevented neuronal loss, and delayed the onset of autoimmune encephalomyelitis suggesting that resveratrol could play an immunomodulatory role in managing multiple sclerosis [165]. Similarly, administration of resveratrol (250 mg/kg/day) for 3 weeks showed therapeutic potential as an adjunct in the treatment of multiple sclerosis by improving motor coordination and balance, mitochondrial function, reducing oxidative stress, and inhibiting NF-κB signaling [166]. In a study of 66 patients with multiple sclerosis who were treated with grape seed capsules for one month it was found that the capsules positively impacted physical and mental functioning, improving quality of life [167]. Given this, polyphenols may have therapeutic potential as an adjunct treatment in multiple sclerosis patients.

## 6. Conclusions

Polyphenols are promising candidates for novel adjunct therapeutic approaches. They can modulate multiple immune system processes and reduce the burden of various diseases such as IBD, allergies and autoimmune disorders. In addition to their demonstrated antioxidant qualities, polyphenols have broad health-promoting effects, due to their ability to modulate inflammation and immune responses. They improve the interplay between immune cells and decrease expression of pro-inflammatory cytokines. Further research is required to clinically validate the therapeutic potential of polyphenols on immunomodulation as well as to explore their interaction with gut microbiota.

## Figures and Tables

**Table 1:** The immunomodulatory effects of polyphenols.

Polyphenols	Signaling Pathways	Immunomodulatory Responses
Curcumin [70,71]	Suppress NF-κB	↓ Bcl-2 in PHA-activated Tcells
		Suppress maturation of DCs
		Inhibit IL-12, IL-8
		↑ IL-4
Resveratrol, Quercetin, Silibinin [72]	Altering PI3K/Akt	↓IL-6 and IL-1
Genistein [71,73]	Activate AMPK	↓ IL-1β, IL-6, IL-8
	Inhibit ROS/Akt/NF-κB	↓COX-2
EGCG [74]	Suppress NF-κB and MAPK	Inhibit Th1 and Th17 differentiation
		↓ Transcription factors (STAT1 and T-bet for Th1, and STAT3 and RORγt for Th17)
		↑ T-reg in lymphoid tissues and central nervous system
Proanthocyanidins Procyanidins [75,76]	Suppress NF-κB and MAPK	↓TNF-α, IL-1β
		Inhibit iNOS and COX-2
Caffeic acid [77,78,79]	Suppress p38 MAPK, JNK1/2 and NF-κB	↓ IL-1β, IL-6, TNF-α
		↓ Monocyte chemoattractant protein (MCP)-1
		Inhibit xanthine oxidase and COX

PHA: Phytohemagglutinin, DC: Dendritic cell, IL: Interleukin, COX: Cyclooxygenase, Th: T helper, STAT: Signal transducer and activator of transcription, NF-κB: Nuclear factor kappa-B, ROS: Reactive oxygen species, TNF-α: Tumor necrosis factor-alpha, MCP: Monocyte chemoattractant protein, iNOS: Inducible nitric oxide synthase, PI3K/Akt: Phosphatidylinositol 3-kinase/protein kinase B, AMPK: Adenosine monophosphate-activated protein kinase, MAPK: Mitogen-Activated Protein Kinase.

**Table 2:** Effect of dietary polyphenols on allergic reaction.

Dietary Polyphenols	Treatment and Duration	Results
**Atopic Eczema or Dermatitis**		
Quercetin (pure isolated polyphenols)	15 human subjects with contact dermatitis. Quercetin applied topically for five days	No change as compared to the control [119]
Cocoa flavanols (catechin, epicatechin, procyanidins) at a lower dose of 27 mg or a higher dose of 329 mg	Ten healthy women consumed a low and high dose.	The higher dose of cocoa drink reduced water loss and improved the blood circulation in the skin [120]
Water extract of whey powder dodder rich in quercetin	Randomized control trial (RCT) study recruited 52 subjects atopic dermatitis recruited for 30 days	Quercetin reduces allergy and inhibits the secretion of the mast cell. Elevate skin moisture and elasticity [121]
Apple condensed tannins (ACT) at a dose of 10 mg/kg	Apple polyphenols were investigated in subjects with atopic eczema for 8 weeks.	Reduced inflammation and itching in disease subjects compared with the control group. ACT has an anti-allergic effect [122]
**Food Allergy**		
Polyphenol enriched extracts or purified epicatechin (1, 0.3 and 0.01%)	Female BALB/c mice treated with polyphenols for 8 days	Epicatechin exhibited a significant anti-allergic effect [123]
Polyphenol-enriched apple extract (>40%)	BALB/c mice treated with an apple extract for 7 weeks	Reduce allergenicity by protein–polyphenol interaction, decrease intestinal mast cell protease and pro-inflammatory genes, diminished cytokine secretion. [105]
Cocoa diet with 0.2% polyphenols	Rats received either a cocoa diet or a standard diet for 4 weeks	Cocoa diet decreased total serum immunoglobulin (Ig)E, Tumor necrosis factor (TNF)-α and interleukin (IL)-10 secretion. No effect on IL-4 synthesis [124]
**Asthma and Rhinitis**		
Drinks containing apple polyphenols at low and high dose (50 mg and 100 mg)	33 subjects having moderate or severe persistent allergic rhinitis treated with apple polyphenols for 4 weeks	Improve sneezing attacks nasal discharger and swelling of the nasal turbinate in the low-dose group and high dose group [125]
100 mg pycnogenol mixture of water-soluble bioflavonoids	76 patients with asthma	Decrease by 15.2% of the specific IgE, whereas IgG1 and IgG4 remained unchanged. Reduced the need for medication [126]
500 mg/day Apple condensed tannins (ACT) and polyphenols	A double-blind comparative study on 36 subjects with rhinitis for 12 weeks	Significant improvement in sneezing scores and nasal discharge inhibited in perennial rhinitis due in the group taking polyphenols treatment [127]

## Data Availability

Not applicable.
